# Hemocompatibility of micropatterned biomaterial surfaces is dependent on topographical feature size

**DOI:** 10.3389/fphys.2022.983187

**Published:** 2022-09-19

**Authors:** Meghan E. Fallon, Hillary H. Le, Novella M. Bates, Yuan Yao, Evelyn K.F. Yim, Monica T. Hinds, Deirdre E.J. Anderson

**Affiliations:** ^1^ Department of Biomedical Engineering, Oregon Health & Science University, Portland, OR, United States; ^2^ Department of Chemical Engineering, University of Waterloo, Waterloo, ON, Canada

**Keywords:** Poly(vinyl alcohol), thrombosis, topography, vascular graft, hemocompatibility, micropattern

## Abstract

Small-diameter synthetic vascular grafts that have improved hemocompatibility and patency remain an unmet clinical need due to thrombosis. A surface modification that has potential to attenuate these failure mechanisms while promoting an endothelial layer is the micropatterning of luminal surfaces. Anisotropic features have been shown to downregulate smooth muscle cell proliferation, direct endothelial migration, and attenuate platelet adhesion and activation. However, the effect of micropatterning feature size and orientation relative to whole blood flow has yet to be investigated within a systematic study. In this work, hemocompatibility of micropattern grating sizes of 2, 5, and 10 µm were investigated. The thrombogenicity of the micropattern surface modifications were characterized by quantifying FXIIa activity, fibrin formation, and static platelet adhesion *in vitro*. Additionally, dynamic platelet attachment and end-point fibrin formation were quantified using an established, flowing whole blood *ex vivo* non-human primate shunt model without antiplatelet or anticoagulant therapies. We observed a higher trend in platelet attachment and significantly increased fibrin formation for larger features. We then investigated the orientation of 2 µm gratings relative to whole blood flow and found no significant differences between the various orientations for platelet attachment, rate of linear platelet attachment, or end-point fibrin formation. MicroCT analysis of micropatterned grafts was utilized to quantify luminal patency. This work is a significant step in the development of novel synthetic biomaterials with improved understanding of hemocompatibility for use in cardiovascular applications.

## 1 Introduction

The development of small-diameter (inner diameter (ID) < 6 mm) synthetic vascular graft biomaterials for the treatment of advanced cardiovascular diseases has been limited by the inability to attenuate common failure mechanisms, such as intimal hyperplasia and thrombosis. While current synthetic biomaterials, such as expanded polytetrafluoroethylene (ePTFE) and Dacron^®^, have shown clinical success for medium- to large-diameter vessels (ID > 6 mm), these biomaterials have poor patency rates when used for small-diameter vascular reconstruction and often result in loss of function (Pashneh-Tala et al., 2016). This early failure has largely been attributed to thrombosis induced by the inadequate establishment of an endothelial layer on the luminal graft surface and poor hemocompatibility at the blood-material interface. As thrombosis is the leading cause of early synthetic graft failure at small diameters, there is a critical need to engineer novel synthetic biomaterials with enhanced hemocompatibility.

Upon graft implantation, the blood-material interface is subject to a dynamic environment, and the interaction between flowing blood and a synthetic surface influences biocompatibility. Upon exposure to blood, biomaterial surfaces can activate the intrinsic, or contact, pathway of coagulation, triggering a cascade of pathophysiological reactions leading from protein adsorption, platelet adhesion, and platelet activation to the generation of thrombin (Braune et al., 2019). These processes ultimately result in the formation of a platelet- and fibrin-rich thrombus on the biomaterial, reducing graft patency and function. Platelet adhesion to the biomaterial surface occurs simultaneously with or immediately following protein adsorption, therefore, making platelet adhesion a key event in thrombus development (Kizhakkedathu and Conway, 2022). Thus, a biomaterial that minimizes platelet adhesion is critical for improving long-term biocompatibility.

Strategies to increase the biocompatibility of synthetic biomaterials have included surface modifications to influence the biological responses at the blood-material interface. Topographical micropatterning is a promising physical surface modification to improve biocompatibility of next-generation synthetic grafts (Chong et al., 2014). Micron-sized ridges and grooves have been shown to downregulate smooth muscle cell growth (Thakar et al., 2009) and encourage *in situ* endothelialization by guiding endothelial migration (Liliensiek et al., 2010), enhance endothelial attachment (Robotti et al., 2014), and improve endothelial function (Gong et al., 2017; Stefopoulos et al., 2017; Dessalles et al., 2021; Fallon and Hinds, 2021). In addition, platelet attachment and activation have been shown to be either unaffected (Cutiongco et al., 2015) or downregulated on anisotropic features compared to unpatterned materials in static culture (Ding et al., 2013; Li, 2013; Ding et al., 2014; Ding et al., 2015). Furthermore, aligned topography has been shown to increase hemocompatibility by decreasing thrombus formation when exposed to whole blood flow (Liu et al., 2013; Cutiongco et al., 2015; Cutiongco et al., 2016; Pohan et al., 2019). Yet, these studies only focused on one size of grating or were performed in a static environment, neglecting to investigate the effect of topographical feature size on platelet attachment in a dynamic, physiological, whole blood environment and lacked consideration of the contact pathway. In addition, topographies under whole blood flow were only oriented parallel to the flow direction, leaving the effect of off-axis flow effects yet to be elucidated. Therefore, anisotropic topographical micropatterning has the potential to mitigate biomaterial-induced thrombosis, but a systematic study of hemocompatibility on micropattern size and directionality when exposed to physiological whole blood flow remains yet to be investigated.

In the present study, we aim to investigate the effects of topographic micropatterning feature size and orientation on vascular graft hemocompatibility under non-anticoagulated, whole blood flow. We hypothesized that micropattern feature size and orientation relative to blood flow would affect the hemocompatibility of a biomaterial within a physiologically and clinically relevant environment. This work is a significant step toward the development of biocompatible synthetic vascular grafts by understanding the effect of micropatterning on thrombus formation within a physiological hemodynamic environment.

## 2 Materials and methods

### 2.1 Ethics approval statement

All animal studies were reviewed and approved by the Oregon National Primate Research Center (ONPRC) Institutional Care and Use Committee (IP00000300). Male olive baboons (*Papio anubis*) were housed and cared for by ONPRC staff according to guidelines set by the National Research Council and the Committee on Care and Use of Laboratory Animals of the Institute of Laboratory Animal Resources.

### 2.2 Manufacturing of poly(vinyl alcohol) films and tubes

#### 2.2.1 Poly(vinyl alcohol) crosslinking solution

Poly(vinyl alcohol) (PVA) films and tubes were manufactured as previously described (Cutiongco et al., 2015; Cutiongco et al., 2016). Briefly, 10% aqueous PVA (Sigma-Aldrich) was crosslinked with 15% w/v sodium trimetaphosphate (Sigma-Aldrich) and 30% w/v NaOH. The PVA solution was then degassed by centrifugation before use in casting films and tubes.

#### 2.2.2 Poly(vinyl alcohol) films

Polydimethylsiloxane (PDMS, Sylgard 184, Dow Corning) molds with various anisotropic topographies were used for casting PVA films. Three anisotropic topographies with various dimensions were studied: (1) gratings with ridge width, groove width, and depth: 2 μm × 2 μm x 2 µm [2 µm], (2) gratings with ridge width, groove width, and depth: 5 μm × 5 μm x 5 µm [5 µm], and (3) gratings with ridge width, groove width, and depth: 10 μm × 10 μm x 10 µm [10 µm]. PDMS molds were treated with air plasma for 1 min. Crosslinking PVA solution was then cast on plasma-treated PDMS molds followed by centrifugation at 1500 RPM for 1 h. After centrifugation, PVA was crosslinked in a cabinet with controlled temperature (20°C) and humidity (60–70%) for 10 days. Micropatterned PVA films were finally demolded using 10X phosphate buffer saline (PBS), 1X PBS, and DI water. Casting onto standard polystyrene petri dishes (Fisher Scientific) was used to fabricate unpatterned, planar PVA [planar].

#### 2.2.3 Poly(vinyl alcohol) tubes

To manufacture PVA tubes, cylindrical molds with patterns for planar, 2 µm gratings, 5 µm gratings, or 10 µm gratings were formed by attaching a thin layer of PDMS with the respective pattern on a rod with an outer diameter of 4 mm. The micropatterned molds were then immersed into the PVA crosslinking solution with sonication for 1 h. The micropatterned molds were then dip-cast repeatedly to manufacture PVA tubes with luminal patterning. The micropatterned PVA tubes were crosslinked in a cabinet with controlled temperature (20°C) and humidity (60–70%) for 3 days. Micropatterned PVA tubes were finally demolded using 10X PBS, 1X PBS, and DI water. Orientations of micropatterns were altered by attaching the patterned PDMS films to the dipping rod at the specified angle.

### 2.3 Scanning electron microscopy imaging

PVA tubes were dried overnight in a 60°C oven before being mounted on aluminum stubs. The micropatterned PVA tubes were then imaged with an environmental scanning electron microscope (SEM, FEI Quanta FEG 250 ESEM) at 20 kV set to high vacuum mode.

### 2.4 *In vitro* hemocompatibility

#### 2.4.1 Washed platelets and platelet-poor plasma

Whole venous blood from healthy volunteers (IRB 1673) was drawn into sodium citrate as previously described (McCarty et al., 2005). Platelet-rich plasma was prepared from whole blood by centrifugation (200*g*, 20 min). For select studies, purified platelets and platelet-poor plasma (PPP) were isolated by further centrifuging platelet-rich plasma (1000*g*, 10 min) in the presence of 0.1 µg/ml prostacyclin. The resulting supernatant was PPP, which was collected and stored for use in the fibrin generation and activated coagulation factor XII (FXIIa) activity assays. The platelet pellet was resuspended in modified HEPES/Tyrode’s buffer with 0.1 µg/ml prostacyclin. Purified platelets were washed 1X by centrifugation (1000*g*, 10 min) and resuspended in HEPES/Tyrode’s buffer for use in the acid phosphatase activity assay.

#### 2.4.2 Factor XIIa activity assay

Pooled PPP was added to a 96-well plate with or without PVA film samples. PPP and glass coverslips (Electron Microscopy Sciences) were used as negative and positive controls, respectively. FXIIa activity generated by the samples were measured using a chromogenic substrate (Chromogenix S-2302, 8 mM, Diapharma Group, Inc.). Absorbance was immediately read on a kinetic cycle at 405 nm every minute for 1 h at 37°C using an Infinite M200 spectrophotometer (Tecan, Männedorf, Switzerland). The rate of the chromogenic substrate cleavage by FXIIa was calculated for each sample over the entire experimental period.

#### 2.4.3 Fibrin generation assay

Pooled PPP was added to a 96-well plate with or without PVA film samples followed by the addition of CaCl_2_ (8.3 mM). PPP and glass coverslips (Electron Microscopy Sciences) were used as negative and positive controls, respectively. Changes in turbidity were immediately measured by reading the absorbance of the solutions at 405 nm every minute for 1 h at 37°C. Fibrin initiation times were quantified at absorbance values 10% above the average baseline absorbance. The rate of fibrin generation was quantified by the maximum slope for each sample.

#### 2.4.4 Static platelet adhesion assay

*In vitro* static platelet adhesion was quantified by measuring platelet acid phosphatase activity (QuantiChrom Acid Phosphatase Assay Kit, Bioassay Systems) as previously described (Bates et al., 2020). Briefly, PVA films were coated in human fibrinogen (50 µg/ml, Enzyme Research Laboratories) for 1 h and washed 3X with PBS. Then, purified platelets (2.0 × 10^8^ platelets/mL) were incubated on PVA samples for 45 min at 37°C and 5% CO_2_ to allow for static adhesion. Glass coverslips (Electron Microscopy Sciences) coated with fibrinogen were used as positive controls. Samples were gently rinsed 3X with sterile PBS to remove non-adherent platelets. Adherent platelets were lysed using 1% Triton-X100 for 5 min at room temperature. Platelet acid phosphatase activity was measured at 405 nm using an Infinite M200 spectrophotometer (Tecan, Männedorf, Switzerland). Number of platelets adhered were calculated according to a standard curve of known platelet solutions (0.0–2.0 × 10^8^ platelets/mL).

### 2.5 Whole blood *ex vivo* testing

#### 2.5.1 Whole blood platelet attachment and fibrin formation under flow

Whole blood *ex vivo* testing of PVA tubular grafts was performed using a chronic, femoral arteriovenous shunt implant in an established non-anticoagulated, non-human primate model (Anderson et al., 2014). In brief, an arteriovenous shunt was surgically implanted between the femoral artery and vein of a juvenile male baboon (14 kg). Single PVA tubes were then incorporated into the shunt as previously described (Anderson et al., 2014). Sections of 9-guage FEP heat shrink tubing (Zeus Industrial Products) were inserted into individual PVA tubes (7 cm long, 4 mm ID). Then, the heat shrink tubes were inserted into Silastic tubing (4 mm ID, Dow Chemical, Midland, MI) and wrapped with parafilm to prevent leakage. Outside the PVA tubes, additional 6-guage heat shrink tubing with parafilm was added to prevent kinking or shifting of the sample. Next, the Silastic tubing was connected to the shunt. Whole blood with autologous platelets and homologous fibrin labelled with indium-111 (^111^In) or iodine-125 (^125^I), respectively, flowed over the tubes in the absence of antiplatelet or anticoagulant therapies. Blood flow rate was measured with a flow probe (Transonic) proximal to the device and controlled to 100 ml/min to recapitulate the mean wall shear rate of 265 s^−1^ of the arterial system at an inner vessel diameter of 4 mm with a manual clamp downstream of the device (Tucker et al., 2009). PVA devices were positioned over a gamma camera (Brivo NM615, General Electric, Boston, MA) to dynamically quantify platelet accumulation on the tubes via measurement of ^111^In radiation. All testing *ex vivo* was performed under equal pressure conditions with a mean arterial blood pressure of 56.4 mm Hg, which was measured during surgical shunt placement. Platelet data were quantified every minute over an experimental period of 60 min total. Rate of platelet attachment was determined within the linear range of dynamic platelet data for each sample. For each sample, the linear range of platelet attachment within the 60 min experimental period was determined. To quantify the rate of platelet attachment, the slope of the linear range was calculated using a linear regression. The amount of fibrin was quantified with a 1480 Wizard gamma counter (PerkinElmer, Waltham, MA) by measurement of ^125^I signal, which was performed approximately 30 days after the experiment to allow for the ^111^In to decay. PVA tubes with topographical patterns (4 mm ID, *n* = 6 for each pattern) were tested. Additional controls were unpatterned, planar PVA tubes (4 mm ID, *n* = 2), clinical ePTFE grafts (4 mm ID, *n* = 2), and collagen-coated ePTFE grafts (4 mm ID, *n* = 2), which served as negative, clinical, and positive controls, respectively. These samples were not compared to the micropatterned grafts statistically, but results were comparable with prior historical controls (Cutiongco et al., 2015; Anderson et al., 2019; Bates et al., 2020; Gupta et al., 2020; Yao et al., 2020).

#### 2.5.2 Micro-computed tomography image analysis

After the 60 min whole blood, *ex vivo* study, tubes were rinsed with PBS and fixed with 3.7% paraformaldehyde (PFA). After 24–72 h of fixation, samples were rinsed and processed for micro-computed tomography (microCT) imaging. Tube lumens were filled with Microfil^®^ (Flow Tech, Inc.) and cured for 2 h at room temperature to render the tube lumens radiopaque, as described previously (Gupta et al., 2020). Positive and clinical control ePTFE grafts were soaked in Lugol’s solution (Sigma) for 16 h to render the thrombus radiopaque. Imaging was performed with a Caliper Quantum FX microCT system (PerkinElmer, Waltham, MA) with settings of a current of 180 μA, voltage of 90 kV, and 60 mm field of view. MicroCT image slices were imported into Amira (FEI, version 5.2.2) to determine average luminal area over the length of the graft for each sample (Supplementary Figure S1). Image analysis was performed by a trained user who was blinded to the sample type, as detailed previously (Gupta et al., 2020).

### 2.6 Statistics

Data are presented as mean ± SD for all studies. Statistical analyses were performed using R (ver. 4.1.2) unless otherwise noted (R Core Team, 2021). Most data were analyzed with a one-way analysis of variance (ANOVA), and a Tukey’s HSD *post-hoc* test was subsequently performed when applicable. *In vitro* fibrin initiation time data were analyzed using a Kruskal–Wallis test followed by a Dunn *post-hoc* test. *In vitro* static platelet adhesion data were analyzed using a two-way ANOVA against the material type and donor followed by a Tukey’s HSD *post-hoc* test. *Ex vivo* platelet quantification data were analyzed in Prism (ver. 9) with a repeated measures ANOVA with a Geisser-Greenhouse correction and a Tukey’s HSD *post-hoc* test. ANOVA assumptions for homogeneity of variances and normality were confirmed with a Brown-Forsythe test and quantile-quantile plots, respectively. Sample sizes (n) correspond to individual PVA films or tubes for *in vitro* or *ex vivo* experiments, respectively. Statistical significance was determined for probability values < 0.05.

## 3 Results

### 3.1 Fabrication of surface topography

**FIGURE 1:**
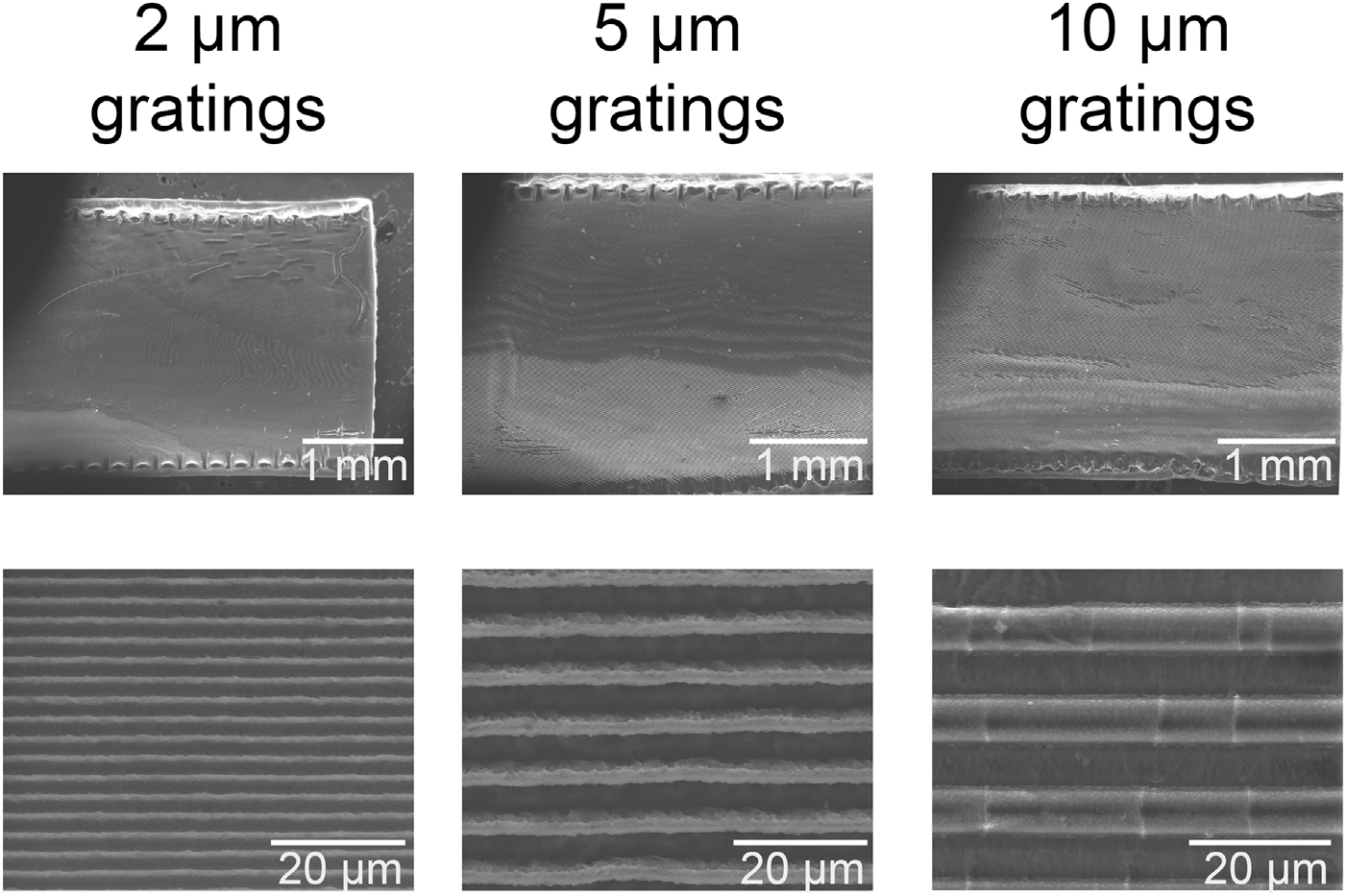
Representative images of topographical features. Scanning electron microscope graphs of micropatterned feature sizes for 2 μm, 5 μm, and 10 μm gratings. Scale bars indicate either 1 mm or 20 µm.

Anisotropic topographical gratings were fabricated on PVA films and the luminal surface of tubes using replica molding. SEM imaging was used to observe the fabrication of features (Figure 1). We observed that the grating patterns on the luminal surface of PVA films and tubes were well-defined. Representative images show highly regular and aligned features after the fabrication process.

### 3.2 *In vitro* FXIIa activity, fibrin initiation time, and rate of fibrin generation

**FIGURE 2:**
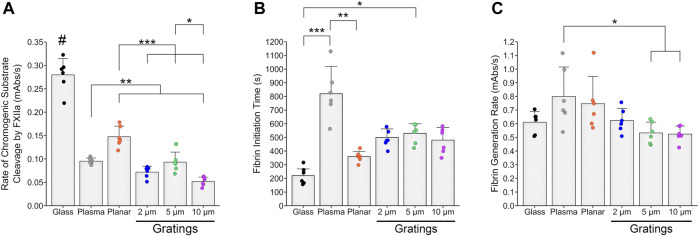
*In vitro* thrombogenicity of micropatterned PVA. Thrombogenicity of PVA samples was determined by FXIIa activity, fibrin initiation time, and fibrin generation rate using static *in vitro* assays compared to glass and plasma as positive and negative controls, respectively. **(A)** FXIIa activity measured by the rate of chromogenic substrate cleavage was significantly reduced for micropatterned PVA compared to planar PVA. FXIIa activity was significantly reduced for the 10 µm gratings compared to the 5 µm gratings. All groups had significantly reduced rate of chromogenic substrate cleavage by FXIIa compared to glass. Plasma was significantly different compared to planar PVA and 10 µm gratings. **(B)** No significant differences for the initiation time to fibrin formation were observed between planar PVA and micropatterned PVA gratings. We did observe a significantly prolonged initiation time to fibrin formation for 5 µm gratings compared to glass. Plasma had a significant increase in initiation time compared to planar PVA and glass due to adsorption of coagulation proteins to the PVA surface. **(C)** No significant differences between the rate of fibrin generation were found between PVA groups or glass. Plasma was observed to have a significantly increased rate of fibrin generation compared to the 5 and 10 µm gratings. Sample size correspond to n = 6 for each experiment. *, **, and *** indicate statistical significance (*p* < 0.05, 0.01, 0.001, respectively). # indicates statistical significance (*p* < 0.0001) compared to all other experimental groups.

Activation of the contact pathway of coagulation was assessed using static *in vitro* assays to determine the interaction between PPP proteins and the biomaterial surfaces (Figure 2A). We found that the rate of chromogenic substrate cleavage by FXIIa was significantly reduced for all micropatterned feature sizes compared to non-patterned, planar PVA. However, 5 µm gratings had a significantly increased cleavage rate compared to the 10 µm gratings. We found that glass, the positive control, had a significantly increased rate of substrate cleavage by FXIIa compared to all PVA samples and plasma, as expected.

Because we saw a decrease in the rate of FXIIa activity by the micropatterns, we sought to study the end effect of the coagulation cascade on *in vitro* fibrin formation. All feature sizes of micropatterned gratings had an increased time to fibrin formation compared to the glass control. However this was only a trend, and only 5 µm gratings had a significant increase compared to glass. The initiation time to fibrin formation was not significant between planar PVA and micropatterned PVA (Figure 2B). There were no significant differences in the rate of fibrin formation between the various micropatterned PVA gratings and planar PVA (Figure 2C).

### 3.3 Static *in vitro* platelet adhesion

**FIGURE 3:**
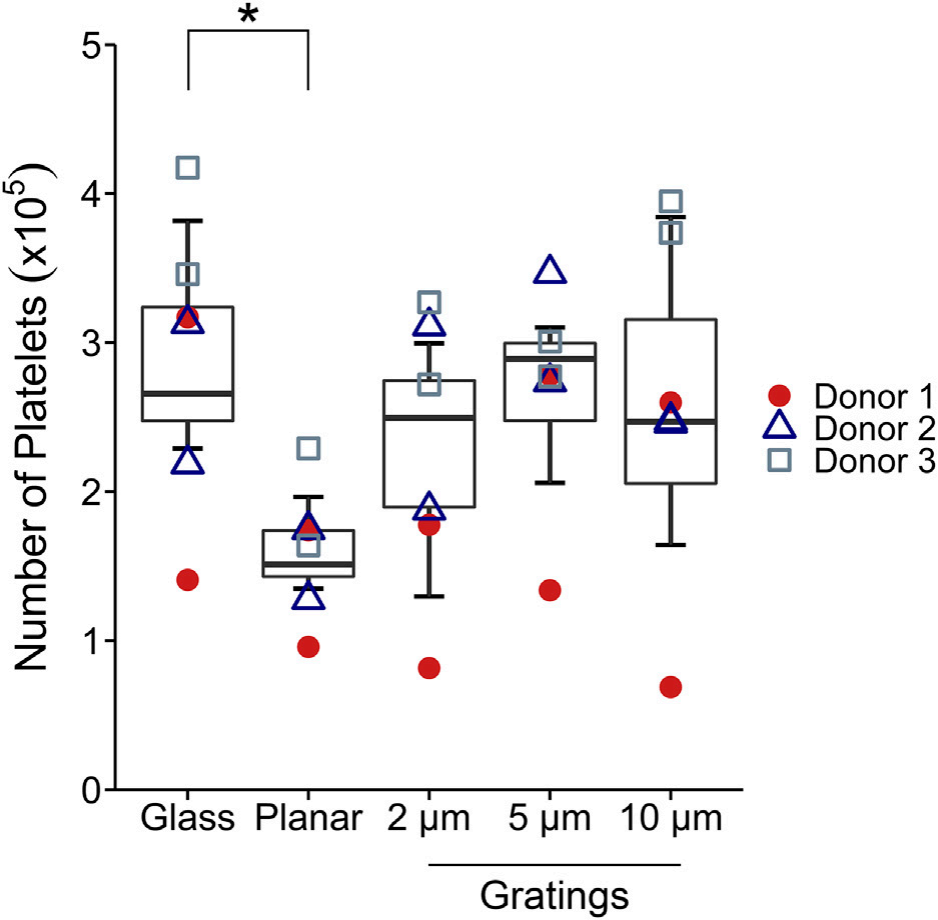
*In vitro* static platelet adhesion. Platelet adhesion after 45 min of static incubation was quantified. Statistical analysis was performed using a two-way ANOVA with factors of the material type and platelet donor with Tukey’s HSD *post-hoc* test (*n* = 6). A significant decrease in the number of platelets adhered was found for planar PVA compared to glass. No significant differences were observed between the micropatterned PVA compared to planar PVA. Significant differences were found between donor 1 and donor 3 (*p* < 0.001). * indicates statistical significance (*p* < 0.05).

We then studied platelet adhesion on micropatterned surfaces using a static, purified platelet *in vitro* assay (Figure 3). Planar PVA had a significant decrease in the number of platelets adhered compared to the positive control of glass. However, no significant differences were observed between planar PVA and PVA with micropatterned gratings. Significant differences were observed between donor 1 and donor 3 (*p* < 0.001).

### 3.4 Effect of micropattern feature size on whole blood dynamic platelet attachment, fibrin formation, and microCT analyses

**FIGURE 4:**
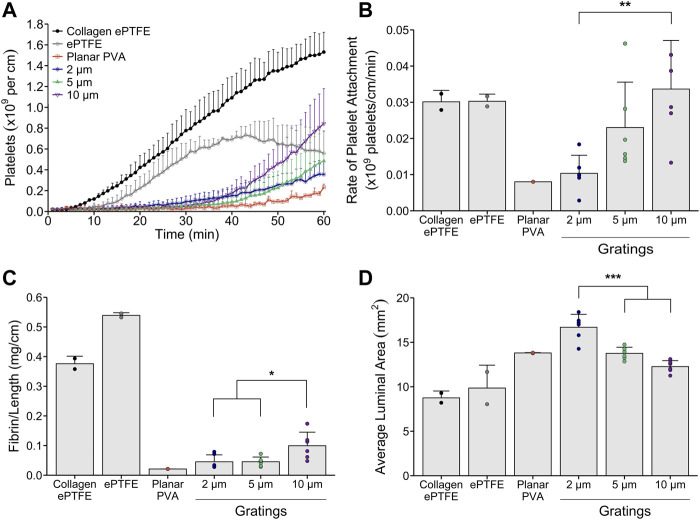
*Ex vivo* thrombogenicity testing of micropattern feature size under physiologic whole blood flow. Tubular micropatterned PVA (2, 5, or 10 µm gratings, n = 6) were tested in an *ex vivo* shunt model to quantify dynamic platelet attachment, rate of platelet attachment, end-point fibrin, and luminal area of the grafts. Micropatterned gratings were oriented parallel to the direction of blood flow. Collagen-coated ePTFE (*n* = 2), ePTFE (*n* = 2), and planar PVA (n = 2) were run as animal controls. **(A)** Platelet accumulation was quantified over 60 min whole blood exposure and normalized to the axial length for all grafts. Statistical analyses were performed only on the micropatterned PVA. There were no significant differences found between the micropatterned PVA. **(B)** The rate of platelet attachment was quantified for the linear range of dynamic platelet accumulation. We observed a trend for increasing rate of attachment as feature size increased. A significant increase in rate was observed for the 10 µm gratings compared to the 2 µm gratings. **(C)** Fibrin data were quantified and normalized to the axial length for all grafts. Significant differences were observed between 10 µm gratings compared to the 2 and 5 µm gratings. **(D)** The average luminal area of the grafts after exposure to whole blood flow were quantified using microCT image analysis. Cross-sectional area per slice of the lumen were analyzed for each graft. Significant differences were observed between 2 µm gratings compared to the 5 and 10 µm gratings. *, **, and *** indicate statistical significance (*p* < 0.05, 0.01, 0.001, respectively).

Using a non-anticoagulated *ex vivo* shunt model, we investigated the effect of feature sizes of micropatterned grafts on dynamic platelet attachment and end-point fibrin formation under physiologic whole blood flow. In the *ex vivo* assessment, micropattern feature size did not significantly affect platelet attachment over time between the micropatterned grafts. However, dynamic platelet attachment trended higher on the 10 µm micropatterned gratings compared to the 2 µm and 5 µm microgratings (Figure 4A). We observed a significantly increased rate of platelet attachment within the linear range of the experimental period for the 10 µm gratings compared to the 2 µm gratings (Figure 4B). Consistently, fibrin formation was significantly increased for the 10 µm micropatterned PVA when compared to both the 2 µm and the 5 µm micropatterned samples (Figure 4C).

We then used microCT imaging to analyze the luminal area of the grafts. Three-dimensional renderings of the luminal area along the length of the samples were generated by selecting the lumen as the area of interest for each image slice. The average luminal patent area for each micropattern size were compared. We found that the 2 µm gratings had a significantly increased average luminal graft patent area compared to the larger 5 and 10 µm patterns (Figure 4D).

### 3.5 Effect of micropattern orientation on whole blood dynamic platelet attachment, fibrin formation, and microCT analyses

**FIGURE 5:**
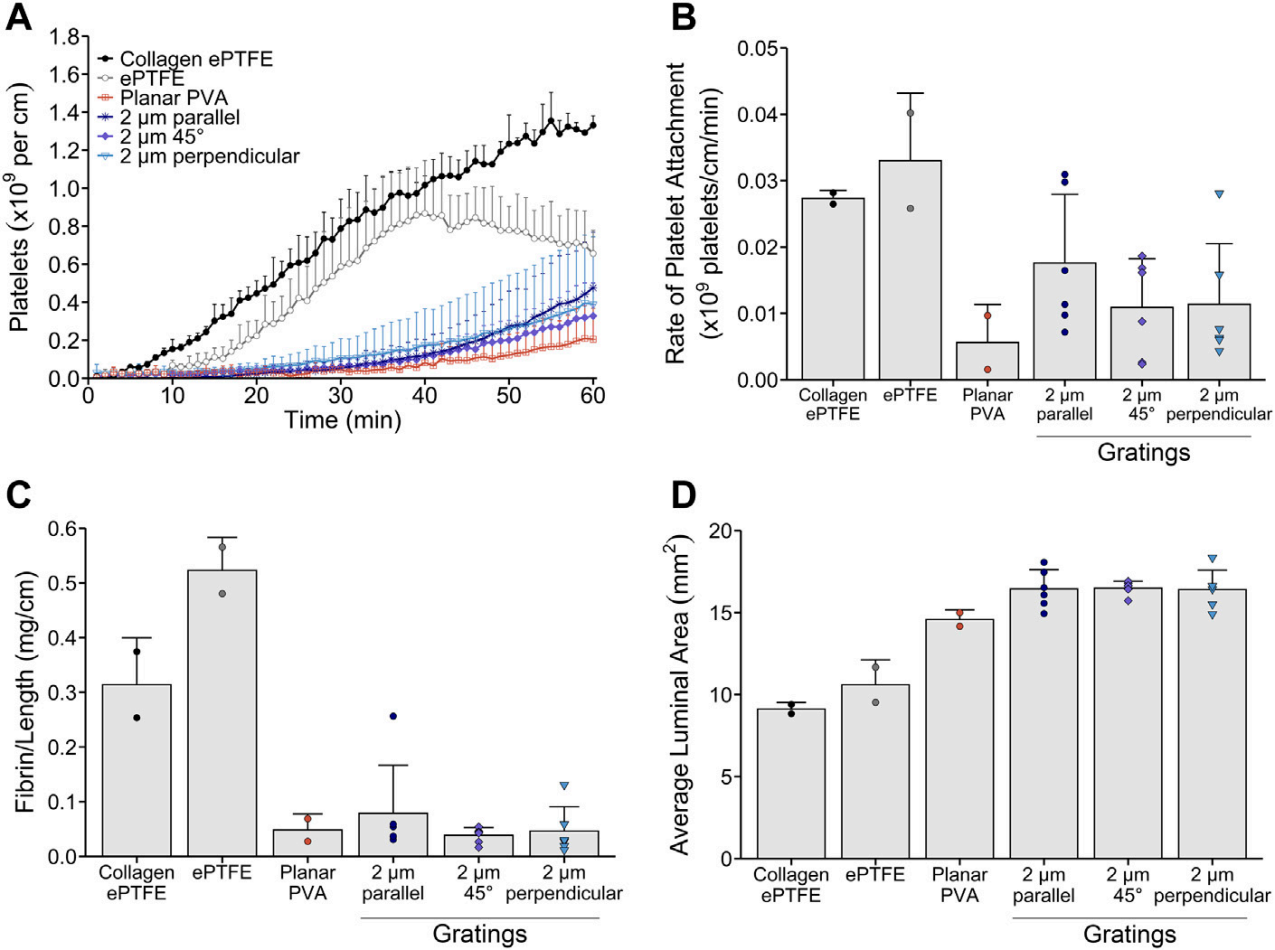
Whole blood, *ex vivo* thrombogenicity testing of micropattern feature orientation relative to physiologic blood flow. Tubular 2 µm micropatterned PVA with gratings oriented either parallel, 45°, or perpendicular relative to whole blood flow (*n* = 6) were tested in an *ex vivo* shunt model to quantify dynamic platelet attachment, rate of platelet attachment, end-point fibrin, and luminal area of the grafts. Collagen-coated ePTFE (*n* = 2), ePTFE (*n* = 2), and planar PVA (n = 2) were run as animal controls. **(A)** Platelet accumulation was quantified over 60 min whole blood exposure and normalized to the axial length for all grafts. Statistical analyses were performed only on the 2 µm grating patterned PVA. There were no significant differences found between the orientations of micropatterned PVA relative to blood flow. **(B)** The rate of platelet attachment was quantified for the linear range of dynamic platelet accumulation. There were no significant differences observed for the rate of platelet attachment between parallel, 45°, or perpendicular orientation of the 2 µm gratings. **(C)** Fibrin data were quantified and normalized to the axial length for all grafts. No significant differences were observed among orientations of the 2 µm gratings relative to blood flow. **(D)** The average luminal area of the grafts after exposure to whole blood flow were quantified using microCT image analysis. Cross-sectional area per slice of the lumen were analyzed for each graft. No significant differences were found.

Since the 2 µm gratings had the least platelet attachment and fibrin formation, we decided to use this feature size to investigate the impact of micropattern feature directionality relative to whole blood flow on dynamic platelet attachment and end-point fibrin formation in the non-anticoagulated *ex vivo* shunt model. The gratings were oriented either parallel, 45°, or perpendicular relative to the direction of blood flow. Orientation of the 2 μm micropatterns relative to blood flow (parallel, 45°, or perpendicular to blood flow) did not significantly affect platelet attachment (Figure 5A), rate of platelet attachment (Figure 5B), fibrin formation (Figure 5C), or average luminal area (Figure 5D).

## 4 Discussion

Small-diameter synthetic vascular grafts have a high rate of failure due to their inherent thrombogenicity, leading to a reduction in graft patency and early device failure. One proposed method to increase hemocompatibility of synthetic materials utilized in vascular graft applications is by establishing an endothelium on the device surface to mitigate material-induced thrombosis and neointimal growth. To accomplish this, topographical micropatterning is a promising physical surface modification that has been shown to direct endothelial cell migration (Liliensiek et al., 2010; Morgan et al., 2012; Dreier et al., 2013; Franco et al., 2013; Potthoff et al., 2014), increase adhesion (Robotti et al., 2014), and improve cell function (Jeon et al., 2015; Shin et al., 2017; Fallon and Hinds, 2021). These studies of endothelial cell responses to topographical micropatterning have all shown superior cell alignment and elongation on subcellular (< 20 μm) feature dimensions relative to endothelial cells. Feature sizes larger than 20 μm have been shown to fail to elongate or align endothelial cells (Vandrangi et al., 2014), supporting the optimization of subcellular feature dimensions for blood-contacting surface engineering. In addition, studies have also shown topographical micropatterning affects platelet attachment (Li, 2013) and activation (Ding et al., 2014), which can influence the thrombotic responses of a material. However, the impacts of varying topographical micropatterned feature size and orientation on contact pathway activation and whole blood-induced thrombosis still need to be fully investigated. One material candidate for vascular grafting is PVA due to the hydrogel’s wide use in biomaterial applications and tuneable mechanical properties. PVA is inherently bio-inert and easily modifiable for enhanced blood compatibility (Chaouat et al., 2008; Alexandre et al., 2015; Jurney et al., 2018; Anderson et al., 2019; Bates et al., 2020; Yao et al., 2020). We hypothesized that the feature size and orientation relative to blood flow would affect the hemocompatibility of PVA. Therefore, we modified the surface of PVA with micron-sized gratings using replica molding. We performed *in vitro* thrombogenicity testing of micropatterned PVA as well as dynamic *ex vivo* whole blood testing to assess the hemocompatibility of the surface modifications. Studies examined both micropattern spacing and orientation.

Thrombosis is a significant contributor to early vascular graft failure and a major concern for blood-contacting materials after implantation, necessitating the design of blood-contacting surfaces to be more hemocompatible. By chemically and/or physically modifying synthetic surfaces, material engineering may be able to increase endothelial growth as well as blood compatibility. However, luminal modifications using chemical surface coatings of biomimicking proteins and small bioactive molecules can result in delamination when exposed to shear stress induced by blood flow. Delamination of the surface coating can expose the synthetic surface directly to blood proteins and cells, prolonging failure mechanisms. Therefore, physical luminal modifications, such as topographical micropatterning, have been of interest in regulating endothelial cell migration and function (Morgan et al., 2012; Dessalles et al., 2021) and in mitigating platelet adhesion and activation (Pocivavsek et al., 2019). Yet, studies investigating the influence of topography on platelet response have varied widely in results, potentially due to the type of underlying material, surface wettability and chemistry, or experimental conditions. We, therefore, sought to experimentally test the hemocompatibility of various feature sizes and orientations on PVA, a promising vascular graft material, under whole blood exposure.

The formation of thrombi on biomaterial surfaces consists of both platelet-mediated reactions (adhesion, activation, and aggregation) and coagulation induced by blood plasma protein interactions with the surface. Proteins can interact with a biomaterial surface and trigger the intrinsic cascade of coagulation. An initial step in protein interaction is FXII converting to the active enzyme, FXIIa, through contact autoactivation, eventually leading to thrombin production and the formation of a fibrin clot. FXII activation is dependent on the surface chemistry, wettability, and surface area of a material (Xu et al., 2014). Topography increases the surface area of the blood-material interface and alters surface wettability, potentially increasing the interaction with plasma proteins and altering thrombogenicity. Therefore, we sought to determine the effect of topographic feature size on *in vitro* thrombogenicity. Using replica molding to modify the surface of PVA with various sizes of anisotropic gratings, we found slight differences within the *in vitro* thrombogenicity assays. We first quantified FXIIa activity on the surfaces to investigate how topography effects the initiation of the contact pathway by FXII activation. We found that all three grating sizes significantly reduced the rate of chromogenic substrate cleavage by FXIIa compared to planar PVA. These results could be explained by the surface chemistry of PVA hydrogel, which has a negatively-charged hydrophilic surface with hydroxyl functional groups (Li et al., 2000; Wiśniewska et al., 2016). These types of surfaces can actively resist protein adsorption due to hydrogen bonding of water to the surface. This interfacial water prevents proteins from displacing the water to adsorb to the hydrophilic surface (Vogler and Siedlecki, 2009). Therefore, because patterning of a hydrophilic surface generally results in an increased wettability, FXII may be less likely to adsorb to the patterned surfaces compared to the non-patterned, planar PVA. This phenomenon may explain our results of lower FXIIa activity for all patterned surfaces compared to planar PVA.

Biomaterial-blood interactions are multifaceted and often require experimentation to understand outcomes. Wettability is often used to predict protein adsorption, and studies have shown that while the addition of topography to planar hydrophilic material surfaces increases the surface area of a material, this surface modification can change the wettability of a material according to the Wenzel and Cassie–Baxter wetting models (Wenzel, 1936; Li, 2013; Liu et al., 2015). The addition of topography to a hydrophilic surface has been shown to increase the hydrophilicity. This increase in hydrophilicity results in a material surface becoming more wettable, allowing water to interact more with a surface due to the increase in contact area and proteins to be less likely to adsorb (Luong-Van et al., 2013). Planar, unmodified PVA is inherently hydrophilic, and the addition of topography to the surface has been shown to change the hydrophilicity in our previous studies (Cutiongco et al., 2015; Cutiongco et al., 2016). Therefore, the possible change in hydrophilicity of the material could account for the decrease in FXIIa activity *in vitro*. Additionally, topography may more directly affect protein adsorption or binding affinity, but this depends on the size and geometry of surface features, surface chemistry, or bulk properties of the material, again requiring scientific testing. Evidence has been shown that micron-sized topographies can influence protein interactions with surfaces (Luong-Van et al., 2013), which could potentially alter blood protein interaction with a surface and potentially affect contact autoactivation. Next, because we saw a significant decrease in FXIIa activity for the topographic surfaces, we then aimed to quantify the end-effect of *in vitro* contact activation by quantifying fibrin generation. We observed no significant differences in the time to initiation of fibrin formation between the PVA materials, but did find that 5 µm gratings had a significantly increased time to fibrin formation compared to glass, with similar trends for 2 µm and 10 µm. We also observed a decreased rate of fibrin generation for 5 µm and 10 µm gratings compared to plasma, but no significant differences within the PVA groups themselves. This could be due to PVA being the common underlying material within these groups.

Platelets are critical to clot composition, especially in arterial blood flow (Chernysh et al., 2020). Studies have previously shown the effects of topography on platelet adhesion and activation *in vitro.* We therefore investigated the effects of topographic feature size on static platelet adhesion within a purified system *in vitro*. The only significant difference observed *in vitro* was between glass and planar PVA while we observed no differences between any of the PVA groups, even with increasing topographic feature size. This is in contrast to other studies, where another group has shown that increasing the space in between ridges led to an increase in both platelet attachment and aggregates in a flat chamber perfusion *in vitro* system (Schieber et al., 2017). Contrastingly, other groups have shown that the addition of topography decreases platelet adhesion and activation compared to planar surfaces, depending on the topographic feature size, ranging from sizes of 0.5–50 µm or 0.5–3.5 µm (Ding et al., 2013; Ding et al., 2014). These differences could be explained by the difference in bulk materials used. Our study utilized PVA, a biologically inert material. This results in PVA not inherently supporting cell attachment, including platelet attachment, to the surface unless further modified (Ino et al., 2013; Huang et al., 2016). Furthermore, we utilized a pre-coating of fibrinogen to mimic the adsorption of blood proteins to a synthetic surface upon *in vivo* whole blood exposure. Fibrinogen adsorption occurs rapidly to biomaterials upon contact with blood plasma and plays a key role in thrombosis on cardiovascular devices (Horbett, 2018). Coating our sample surfaces prior to incubating platelets on the surface to mimic this physiological process could also account for differences in our results compared to other studies. Additionally, studies have investigated electrospun fibrous scaffolds and found a decrease in platelet adhesion and activation when compared to smooth substrates (Lamichhane et al., 2016). Another study by Yu *et al.* investigated the effect of varying aligned electrospun fiber diameter on *in vitro* hemocompatibility (Yu et al., 2020). They found that while the various aligned fibers exhibited reduced platelet attachment and a rounded morphology, the anticoagulation properties improved as fiber diameter decreased from the micron-scale to the nano-scale. While our study only focused on the micron-scale, this trend of improved hemocompatibility with decreasing features size is in agreement with our results.

While we saw slight improvements in thrombogenicity of micropatterned surfaces compared to planar PVA *in vitro*, these assays were performed with purified, static systems. While static *in vitro* assays are useful for the study of biomaterial hemocompatibility, they fail to fully recapitulate the complex hemodynamic field to which clinically implanted vascular graft biomaterials are exposed (Ghista and Kabinejadian, 2013). Thrombus formation is highly dependent on the local flow conditions produced by pulsatile blood flow. The effects of flow profiles and shear rates directly impact key factors involved in biomaterial-induced thrombosis, such as protein accumulation, complement activation, platelet adhesion and activation, and interaction with other blood constituents, such as red blood cells (Hathcock, 2006; Casa et al., 2015; Hong et al., 2020). Model systems should consequently allow evaluation of novel biomaterial surface modifications with whole blood under physiologic flow conditions to fully investigate blood-material interactions. Therefore, we aimed to assess thrombogenicity of the micropatterns in a more physiologically-relevant environment using an *ex vivo* non-human primate shunt model. This established model uses non-anticoagulated, whole flowing blood to examine material-induced thrombosis under hemodynamically relevant conditions (Hanson et al., 1980; Crosby et al., 2013). Our model better predicts clinical performance and physiological effects of materials compared to *in vitro* isolated component analyses. We first investigated the effect of micropattern grating size oriented parallel to the blood flow and found no significant differences within the micropatterned groups. Previous work from our group has shown that topographical micropatterned PVA grafts do not significantly increase platelet attachment compared to planar PVA (Cutiongco et al., 2015; Cutiongco et al., 2016; Pohan et al., 2019). However, we did observe a trend of increased platelet attachment for increasing grating sizes. We observed a significant increase in the rate of platelet attachment over the linear range of dynamic platelet accumulation for 10 µm gratings compared to the 2 µm and 5 µm gratings. This is in contrast to the clinical control, ePTFE, which had a plateau of platelet attachment beginning around 35 min of whole blood exposure. Dynamic platelet attachment on PVA samples consistently increased with time over the 60 min experimental period. Future work should investigate platelet attachment over longer periods of time. Comparatively, we found a significant increase in end-point fibrin formation on the 10 µm gratings compared to both the 2 µm and the 5 µm gratings. This is reflected in the microCT data showing the 2 µm gratings had a significantly larger average luminal area compared to the other gratings. A larger average luminal area corresponds to less thrombus within the graft and to an increased graft patency. Based on calliper measurements of the inner diameter of each sample, we assumed a perfect circular graft cross-sectional area. The average luminal area of each graft based on microCT analyses was subtracted to estimate patency at the end of the study. Micropatterned PVA grafts had an average patency of 99%, 94%, and 86% for 2, 5, and 10 µm gratings, respectively. In comparison, the negative, clinical, and positive controls of planar PVA, ePTFE, and collagen-coated ePTFE, respectively, resulting in an average patency of approximately 100%, 91%, or 67%, respectively. These differences could be due to the size of the topographic features. Platelet diameter and size at resting state is approximately 2 µm. Thus, platelets are limited to the top of the 2 µm grating ridges and not the grooves, resulting in less contact area with which to interact. At scales >2 μm, platelets are able to adhere within the grooves, leading to an increased attachment. Additionally, whole blood is composed of many types of cells. At grating sizes of 10 μm, red blood cells have the ability to interact and attach within the grooves, which could account for the decrease in average luminal area for the larger feature sizes (Keegan et al., 2016). Additionally, one proposed theory of fluid shear stress differences between the top and bottom of microstructures could affect platelet adhesion due to a higher shear difference at the top of the microstructure, resulting in a reduced contact time between platelets and the surface. At the bottom of the microstructures, the theory also proposes that blood velocity reduces and results in a trapped blood layer, altering platelet attachment and adhesion depending on the interspacing and size of the microstructures (Pham et al., 2016). The fluid dynamics and boundary layer changes introduced by the topography may account for differences in platelet adhesion results between the *in vitro* and *ex vivo* studies.

Because we observed minimal platelet attachment, minimal fibrin formation, and maximum luminal area for the 2 µm gratings, we selected this feature size to study the effect of pattern orientation relative to whole blood flow on thrombosis. Surprisingly, we found no significant differences in platelet attachment, rate of platelet attachment, fibrin formation, or average luminal area between all orientations. All 2 µm grating orientations had approximately 100% patency by the end of the study compared to the negative, clinical, and positive controls of planar PVA, ePTFE, and collagen-coated ePTFE, respectively. These controls resulted in a patency of either 97%, 90%, or 81% by the end of the study, respectively. These data show that 2 µm gratings do not affect thrombosis with off-axis flow effects relative to blood flow. This could be due to the size of the gratings on the surface that is interacting with platelets, and the results of this study most likely would not translate to larger grating sizes. The lack of change in dynamic platelet adhesion and fibrin deposition regardless of the 2 µm grating orientation relative to blood flow indicates that this surface modification could be useful for cell-based motivations without affecting the thrombotic response of the bulk material, but additional grating sizes and configurations remain to be tested. Therefore, 2 µm gratings could induce *in situ* endothelialization while mitigating thrombosis. While this study did not directly investigate endothelialization on 2 µm gratings, our previous study using an *in vivo* rat abdominal aorta model showed RECA-1 positive infiltration of endothelial cells on the graft lumen after implantation of 20 days of 2 µm grating micropatterned PVA (Cutiongco et al., 2015).

Ultimately, the work presented within this study supported our hypothesis that micropattern feature size affects hemocompatibility of synthetic surfaces while the orientation of 2 µm gratings did not. We showed that micropattern feature size has minimal effects on *in vitro* thrombogenicity and platelet adhesion within purified, static assays. We have shown that increasing feature size does not affect platelet attachment over a 60 min dynamic experimental period but does affect platelet attachment rate, end-point fibrin formation, and average graft luminal area. We also have shown that orientation of 2 µm gratings relative to whole blood flow has minimal effect on *ex vivo* thrombosis and does not significantly affect platelet attachment, platelet attachment rate, end-point fibrin formation, or average luminal area of the micropatterned grafts. Limitations within this study include the short experimental period in which thrombosis is studied as well as the lack of investigation of the effects of larger grating size orientation relative to blood flow. Future work will increase the experimental period to determine the effects on thrombosis after 60 min and should also investigate the effect of various orientations of larger micrograting sizes relative to blood flow. Additionally, an implant model will be used in future work to study neointimal hyperplasia formation and the endothelialization of various micropatterned dimensions and orientations *in vivo*. Lastly, future work will also study the impact of additional chemical modifications with topography to investigate surface-modified PVA as a novel synthetic vascular device. The results reported herein support topographical micropatterning as a promising surface modification that maintains synthetic material hemocompatibility.

## Data Availability

The raw data supporting the conclusion of this article will be made available by the authors, without undue reservation.
