# Supplementary figures and images for: Hemocompatibility of micropatterned biomaterial surfaces is dependent on topographical feature size
Source: Front Physiol. 2022 Sep 19;13:983187. doi: 10.3389/fphys.2022.983187 (PMC9527343; doi:10.3389/fphys.2022.983187)

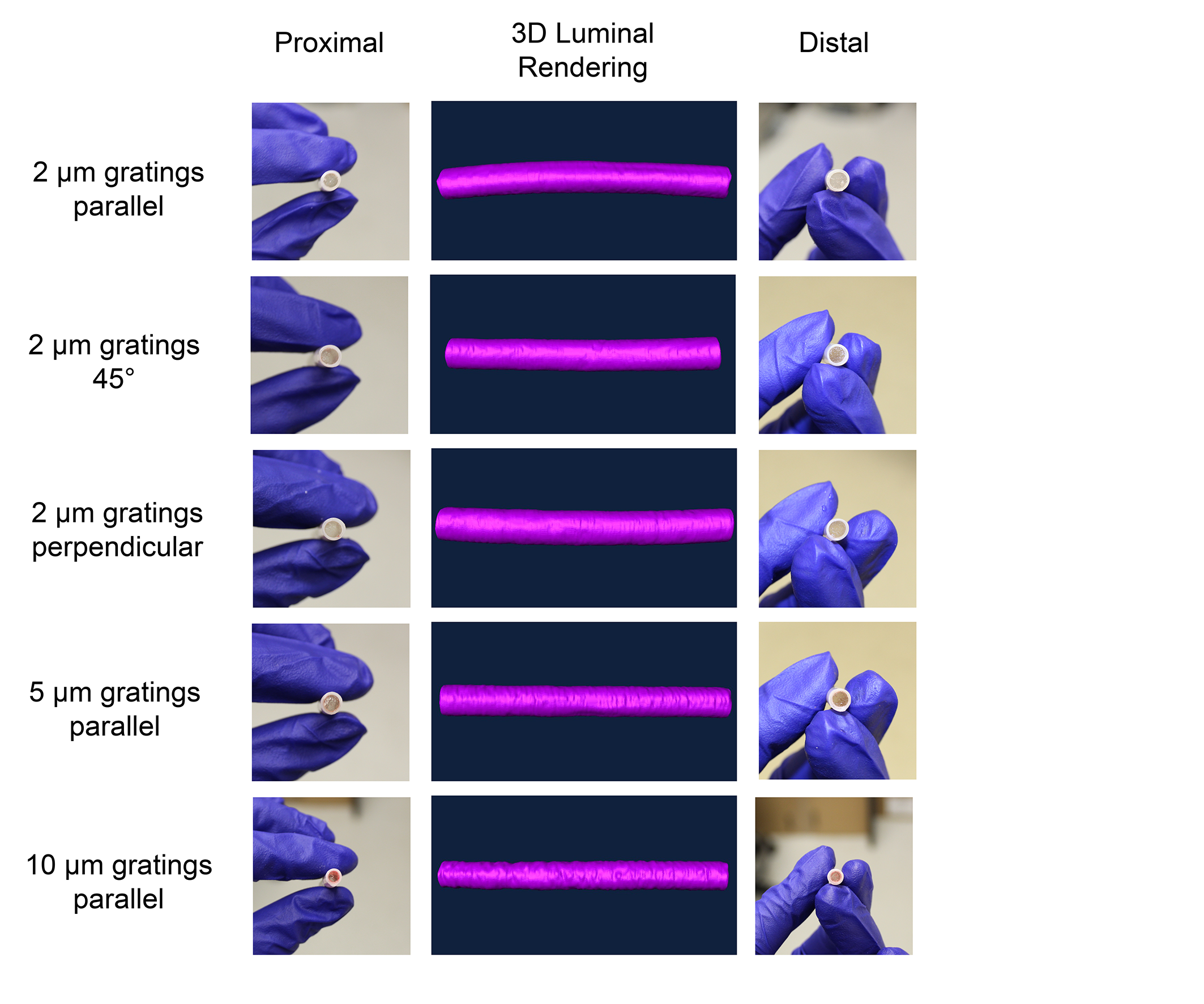

Supplement: Supplementary file 1 [file Image1.TIF]
